# Near-Infrared Spectroscopy as an Analytical Process Technology for the On-Line Quantification of Water Precipitation Processes during Danhong Injection

**DOI:** 10.1155/2015/313471

**Published:** 2015-12-29

**Authors:** Xuesong Liu, Chunyan Wu, Shu Geng, Ye Jin, Lianjun Luan, Yong Chen, Yongjiang Wu

**Affiliations:** College of Pharmaceutical Sciences, Zhejiang University, Hangzhou 310058, China

## Abstract

This paper used near-infrared (NIR) spectroscopy for the on-line quantitative monitoring of water precipitation during Danhong injection. For these NIR measurements, two fiber optic probes designed to transmit NIR radiation through a 2 mm flow cell were used to collect spectra in real-time. Partial least squares regression (PLSR) was developed as the preferred chemometrics quantitative analysis of the critical intermediate qualities: the danshensu (DSS, (R)-3, 4-dihydroxyphenyllactic acid), protocatechuic aldehyde (PA), rosmarinic acid (RA), and salvianolic acid B (SAB) concentrations. Optimized PLSR models were successfully built and used for on-line detecting of the concentrations of DSS, PA, RA, and SAB of water precipitation during Danhong injection. Besides, the information of DSS, PA, RA, and SAB concentrations would be instantly fed back to site technical personnel for control and adjustment timely. The verification experiments determined that the predicted values agreed with the actual homologic value.

## 1. Introduction

Danshen and Honghua are two traditional herbal medicines commonly used in China for the treatment of cardiovascular diseases. Danhong injections are prepared from aqueous Danshen (*Salvia miltiorrhiza *Bunge) and Honghua (*Carthamus tinctorius *L.) extracts. Danhong injections are applied to activate circulation of the blood and resolve stasis to promote regeneration [1], and the formula is applied extensively to tens of millions of patients for the prevention and treatment of coronary artery disease [2] in clinics in China. The crude extracts of Danshen and Honghua contain both fat-soluble and water-soluble compounds. The fat-soluble compounds, such as tanshinone, are primarily antibacterial and antiphlogosis [3]. Meanwhile, the water-soluble ingredients, such as phenolic acids, are the medicinal components in the Danhong injection and promote blood circulation and resolve stasis to promote regeneration [4]. The Danhong injection quality depends on separating and purifying the crude Danshen and Honghua extracts. The industrial production of the Danhong injection uses alcohol precipitation initially to purify the crude extract. This process removes some macromolecular substances such as proteins, polysaccharides, tannins, and pigments [5]. However, certain fat-soluble compounds, primarily tanshinones, are removed via subsequent water precipitations [6].

As a separation technology for producing botanical medicines, water precipitation has been researched less than ethanol precipitation. This work uses water precipitation to purify the ethanol precipitation solution for a Danhong injection to describe its separation characteristics. During the water precipitation process, several active ingredients more or less precipitated with impurities via encapsulation. To data, few studies have examined the loss of active ingredients, such as danshensu (DSS), protocatechuic aldehyde (PA), rosmarinic acid (RA), and salvianolic acid B (SAB) [7, 8]. Danshensu [3-(3,4-dihydroxyphenyl)-2-hydroxy-propanoic acid; DSS] is the major biologically active and water soluble component in Danshen. In vitro and in vivo experiments have demonstrated that DSS has antioxidant, antiatherosclerotic, and endothelial cell protective effects [9, 10]. PA inhibits migration and proliferation of vascular smooth muscle cells and intravascular thrombosis [11]. RA prevents against memory deficits in ischemic mice [12] and has potent antiviral activity against enterovirus 71 infections [13]. SAB has antipulmonary fibrotic activity [14] and attenuates lung inflammation induced by cigarette smoke in mice [15]. Guaranteeing the final Danhong injection quality requires water precipitation research. Detection using on-line, real-time measurements would greatly improve the process efficiency for water precipitation.

Radiation in the near-infrared (NIR) energetic range excites overtone and combination vibrations in the sample material, revealing a high content of chemical as well as physical information at the same time dispersed in the whole spectral area [16]. NIR region covers wavelengths from 780 nm up to 2.5 *μ*m and mainly reflects the overtones and combinations of fundamental vibrations of C-H, N-H, O-H, and S-H bond in organic molecules [17, 18]. Multivariate data evaluation methods were employed to extract the NIR spectral features and to investigate the correlation between the spectral data and the concentration (content) variables measured by the reference assays [19, 20]. Together with multivariate data evaluation methods, NIR spectroscopy has become a popular process control tool with a broad range of applications for precise material characterization [21]. In 2004, the Food and Drug Administration (FDA) issued a guidance document to the pharmaceutical industry regarding the implementation of process analytical technology (PAT) [22]. NIR spectroscopy was applied to develop a fast and reliable quality control system for a pharmaceutical substance to support information obtained through PAT surveillance of its manufacturing process. NIR spectroscopy with multivariate classification as a PAT production control supplement was applied to reliably determine the quality of the end product at minimum measuring effort and to at least partially replace laborious, conventional analysis methods in a long term [23].

As a commonly used PAT tool, NIR spectroscopy is a fast and nondestructive technique; it requires no sample preparation and will not produce any waste products. NIR spectroscopy can be considered as a powerful tool for raw material testing, product quality control, and process monitoring [24–26] in the pharmaceutical industry and, moreover, it has gained wide acceptance by pharmaceutical manufacturers and regulatory agencies. NIR spectroscopy has been investigated and successfully applied to on-line detection during the alcohol precipitation processes for Danhong injections [27]. The variations between the NIR spectra and intermediate quality attributes were investigated. The results indicate the NIR models were suitable for the real-time on-line of alcohol precipitation. A method [28] based on high-performance liquid chromatography combined with chromatographic fingerprint analysis was developed to quantitatively analyze ten Danhong injection components. This outstanding and precise method simultaneously detects multi-indexes, including DSS, PA, RA, and SAB. However, investigation of the variations in the important intermediate quality attributes during water precipitation processes for Danhong injections was not performed.

This paper used NIR spectroscopy for the on-line monitoring of water precipitation processes for Danhong injections. As an analytical process technology, NIR spectroscopy was tested to monitor the quality during water precipitation. DSS, PA, RA, and SAB, the principal bioactive components in the Danhong injection, were selected as the modeling parameters [29].

## 2. Materials and Methods

### 2.1. Materials

The Danhong alcohol precipitation solution was provided by Heze Buchang Pharmaceutical Co., Ltd. (Heze, Shandong, China). The PA standard was purchased from the National Institute for the Control of Pharmaceutical and Biological Products (Beijing, China). The DSS, RA, and SAB standards were all purchased from Chengdu Must Bio-technology Co., Ltd. (Chengdu, China). The standard purities were above 98%. Other reagents were of analytical grade.

### 2.2. On-Line NIR Monitoring Device

**Figure 1:**
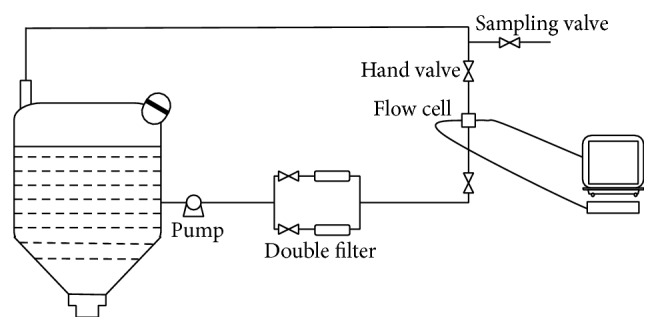
Scheme for the on-line NIR spectroscopy detection device.

The on-line NIR monitoring device for the water precipitation process is shown in Figure 1. This device consisted of a Bruker Matrix-F Fourier transform NIR spectrometer (Bruker Optic Inc., Germany), peristaltic pump, duplex filters (100-mesh sieve for removing deposit and reducing velocity), two hand valves, and one sampling valve. The on-line measurements used two fiber optic probes designed to transmit NIR radiation through a 2 mm flow cell (Solvias, Germany) connected to the Matrix-F Fourier transform NIR spectrometer. When the water precipitation solution passed through the flow cell, the flow rate, solid impurities, and bubbles significantly influenced the collected NIR spectra [30]. However, avoiding such influences is difficult for in-line methods, for example, inserting the NIR probe into the reactor, as described in [31]. To eliminate these influences during this study, the solution was passed through the duplex filters before entering the circulation loop to ensure an even distribution of the chemical components in the reactor. The peristaltic pump maintained the solution flow rate through the flow cell and reduced the effect of the flow rate on the NIR spectra. Hand valves and sampling valves were designed for sampling during the NIR spectra collection.

### 2.3. On-Line Collection of NIR Spectra and Samples

Spectra were collected from 4500 to 12000 cm^−1^ with a resolution of 8 cm^−1^. Each spectrum was collected in the absorbance mode using the average of 32 scans. To capture the variation during water precipitation, NIR spectra were collected every 30 seconds in real-time. However, samples were added every 2 minutes via the sampling valve. Meanwhile, the frequency parameter for the peristaltic pump was set to 20.00 Hz to continuously pump water precipitation solution through the flow cell. At the moment, the flow rate in the flow cell was 360 L/h.

The water precipitation experiment was performed five times, and 146 samples were collected. The first batch was used for the preexperiment, the fifth batch was used as a prediction set for validation, and remaining batches were identified as calibration sets for the model. Number of samples in calibration set and in validation set was 89 and 32, respectively. In total, all spectral pretreatments and chemometrics analyses were performed using OPUS software (version 7.0, Bruker, Germany).

### 2.4. High Performance Liquid Chromatography (HPLC) Analysis Method

This paper used HPLC as the reference method to simultaneously quantify the DSS, PA, RA, and SAB.

#### 2.4.1. HPLC Conditions

**Figure 2:**
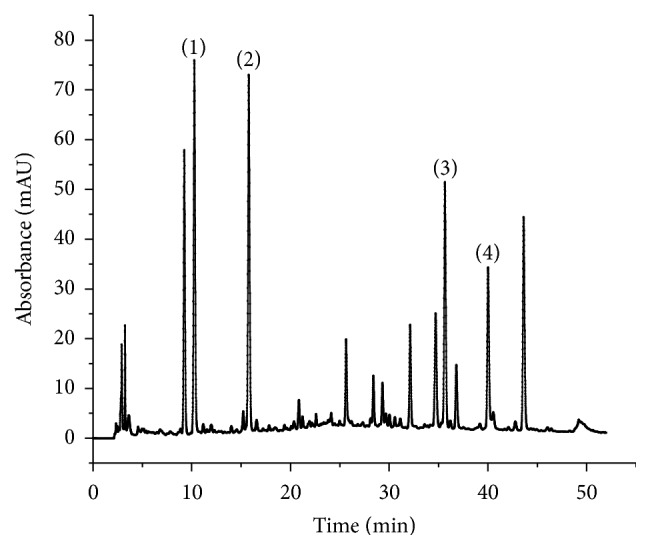
Typical chromatogram of sample. (1) DSS, (2) PA, (3) RA, and (4) SAB.

The chromatographic analysis was performed on an Agilent 1200 HPLC system (Agilent Technologies, USA) equipped with a diode array detector (wavelength range from 190 nm to 949 nm) and an Agilent ChemStation software used for recording chromatograms. All samples were separated on an Agilent Eclipse-C18 analytical column (4.6 mm × 250 mm, 5 *μ*m particle size) at 35°C. The mobile phase was a mixture of (A) methanol and (B) aqueous solutions containing 0.5% (v/v) formic acid. The gradient elution procedure was as follows: initial 9% (A); 0–20 min, linear change from 9% to 39% (A); 20–36 min, linear change from 39% to 47% (A); 36–39 min, linear change from 47% to 90% (A); hold at 90% (A) for 39–45 min. The reequilibration duration between individual runs was 10 min. The mobile phase flow rate was fixed to 1.0 mL/min. The detection wavelength was 280 nm at 0–13 min, was converted to 403 nm at 13–21 min, and reconverted into 280 nm at 21–45 min. The samples during this research were diluted before centrifuging at a rotating speed of 1500 rpm for 10 min, the supernatant was filtered with a 0.45 *μ*m Nylon microfiltration membrane (Beijing Envta Technology CO., Ltd., the location of the producer is room 210 C, Jiaxin Business Building, No. 59, Annei street, Dongcheng district, Beijing, China), and 5 *μ*L of the filtrate was injected into the HPLC system for analysis. One chromatogram including peak assignment is shown in Figure 2.

#### 2.4.2. HPLC Method Validation

The developed HPLC method was validated based on its linearity, precision, stability, and accuracy. Parameters of HPLC method validation for DSS, PA, RA, and SAB were presented in Table 1. Standard curves were obtained from the linear regression for the peak area versus the respective concentrations for the standard analytes. The regression equations and correlation coefficients (*R*) were *y* = 3.241*x* − 1.152 (*R* = 0.9999), *y* = 22.697*x* − 2.542 (*R* = 0.9999), *y* = 8.554*x* − 1.459 (*R* = 0.9999), and *y* = 5.984*x* − 64.335 (*R* = 0.9999) for DSS, PA, RA, and SAB, respectively. Good linear relationships were achieved for the range from 9.84 to 147.6, 1.13 to 22.68, 4.83 to 57.98, and 52.80 to 1056 *μ*g/mL for DSS, PA, RA, and SAB, respectively. In this calibration range, six standards (concentrations) were applied and two consecutive injections were performed.

To determine the method repeatability, a sample (number 12 from the second batch) was randomly selected and analyzed by consecutively injecting six needles under the above HPLC conditions. The relative standard deviation (RSD) in the peak areas for DSS, PA, RA, and SAB was 0.18%, 1.08%, 0.46%, and 0.10%, respectively. These results suggest that the instrument precision was acceptable. Additionally, the stability was tested by analyzing the same sample every 2 h for 12 h at room temperature. The RSD in the peak areas for DSS, PA, RA, and SAB were 0.88%, 0.38%, 0.60%, and 0.17%, respectively, which indicates that samples were stable for 12 h. The accuracy was evaluated using a recovery test via the standard addition method at three concentrations. Accordingly, previously analyzed samples (number 12 from the second batch) were spiked with DSS, PA, RA, and SAB to 0.5, 1.0, and 1.5 times their amount in the sample, respectively. The average recoveries were 101.2%, 98.2%, 99.2%, and 100.8% for DSS, PA, RA, and SAB with RSD below 2.0%. The above validation data indicates the developed HPLC method was acceptable for determining DSS, PA, RA, and SAB.

### 2.5. Chemometrics and Data Analysis

The spectral data were manipulated by identifying the usable spectral regions selecting appropriate preprocessing methods and correlating to the quantitative HPLC data with PLSR to develop the calibration models. During this work, the raw spectra were pretreated using derivatives, straight line subtraction (SLS), vector normalization (VN), standard normal variate (SNV), and multiplicative scatter correction (MSC). Derivatives, including the first derivative (1st Der) and second derivative (2nd Der), were introduced to remove any spectral baseline drift [32]. The SLS corrected the baseline. The VN and MSC are commonly used to eliminate irrelevant information in the spectra from unknown sources such as surface irregularities, distance variation of sample, and detector [33]. Specifically, a spectrum undergoes VN by subtracting the average intensity, and the MSC corrects any multiplicative effects due to scattering via the linear transformation of each spectrum. The SNV was also considered as a scatter correction method [34]. The partial least squares regression (PLSR) helped correlate the pretreated spectral data to the indicator contents to construct the calibration models [35]. To avoid under- or overfitting, the optimum number of latent variables (LVs) in the PLSR models was determined via the Leave-One-Out cross-validation method [36]. The PLSR computations were performed using the OPUS software (version 7.0, Bruker, Germany).

The predictive capabilities of the developed PLSR models were estimated via the coefficient of determination (*R* ^2^), root mean square errors of calibration and prediction (RMSEC and RMSEP, resp.), relative standard errors of calibration and prediction (RSEC and RSEP, resp.), root mean squares error of cross-validation (RMSECV), and ratio of prediction to deviation (RPD) for the calibration set. An excellent model generally has low RMSEC, RMSEP, and RMSECV; high *R* ^2^; and a small difference between the RMSEC and RMSECV. Moreover, the RMSEP value should be close to the RMSEC value.

## 3. Results and Discussion

### 3.1. Results of HPLC Determination

**Figure 3:**
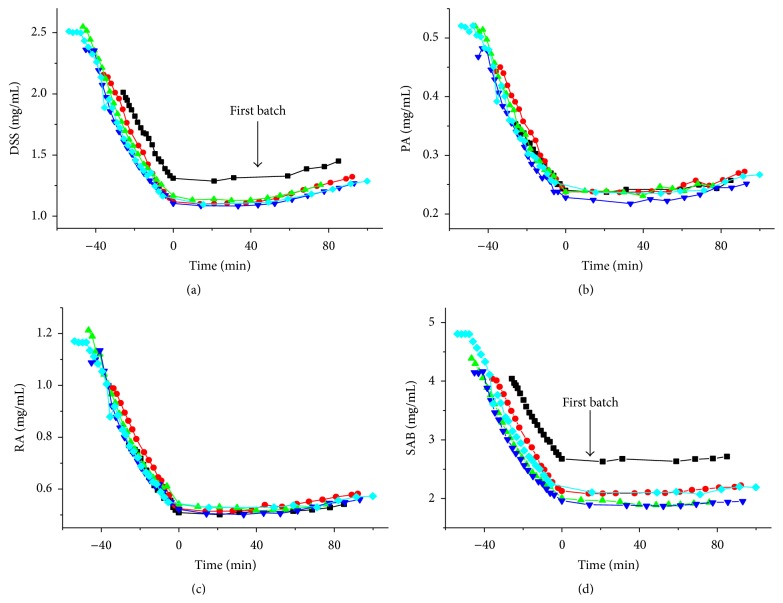
DSS (a), PA (b), RA (c), and SAB (d) concentrations measured by HPLC.

The dynamic course of the DSS, PA, RA, and SAB concentrations during the water precipitation process for Danhong injection is shown in Figure 3. The time evolution curves from the water precipitation process were divided into two phases based on the zero minute. One phase referenced the water-adding stage before 0 min, while the other was the thermostatic stage from 0 to 100 min. For the five batches, the first batch began its water precipitation process two days later than the others. Thus, the first batch was different from the other four batches for DSS and SAB and indicated the water precipitation process should be performed immediately after the alcohol precipitation. The DSS, PA, RA, and SAB concentrations at the end of the water precipitation are summarized in Table 2. The average DSS, PA, RA, and SAB concentrations for the four parallel batches were 1.28, 0.260, 0.564, and 2.07 mg/mL, respectively. The RSD values were 2.19%, 4.54%, 3.13%, and 7.35% for the DSS, PA, RA, and SAB concentrations, respectively.

### 3.2. Selection of NIR Spectral Regions

**Figure 4:**
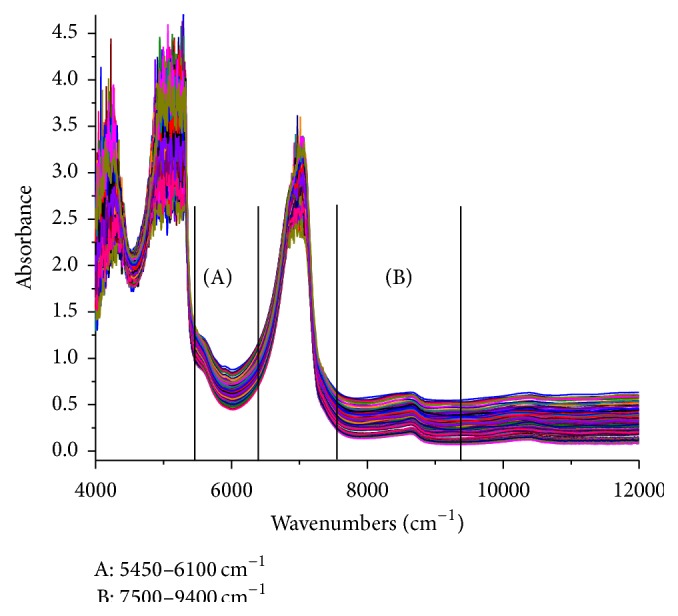
Raw NIR spectra for the Danhong injection water precipitation process.

**Figure 5:**
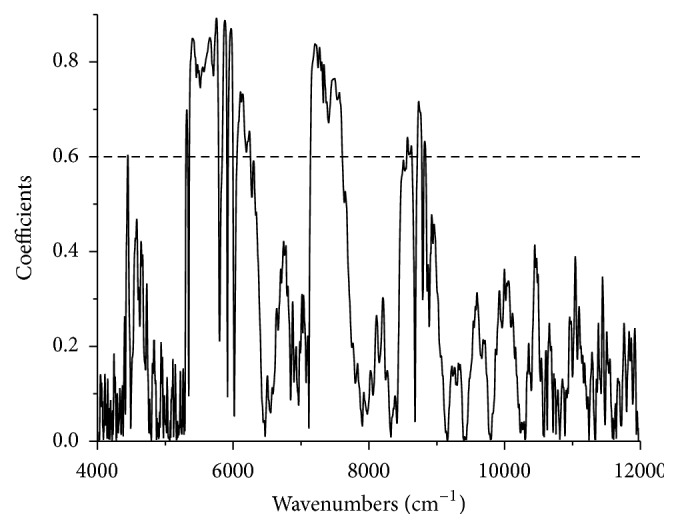
Correlation coefficients for the 1st Der NIR spectroscopy.

Raw NIR spectra for the water precipitation solutions ranging from 4000 to 12000 cm^−1^ were acquired during the monitoring period as shown in Figure 4. The 4000 to 4600 cm^−1^ region had some noise, which was caused by the optical fiber absorption [37]. Simultaneously, the noise was significantly enhanced from 4600 to 5450 cm^−1^ and 6100 to 7500 cm^−1^ (strong signals from water) [38] due to the flow rate of the water precipitation solution through the flow cell. Additionally, the region from 9400 to 12000 cm^−1^ exhibited low intensities and a low signal-to-noise ratio [39]. Furthermore, spectral regions with absorbances equal to or high than 1.5, such as 4000 to 5450 cm^−1^, were considered inappropriate for the spectral analysis due to their zero transmissivity, and they were considered saturated [36]. Hence, removing the abovementioned regions may improve the calibration model accuracy. The remaining regions, 5450–6100 cm^−1^ and 7400–9500 cm^−1^, were utilized for the DSS, PA, RA, and SAB models. To confirm the choice of spectral region was valid, the correlation coefficients for the spectra were investigated as shown in Figure 5. The coefficients for the selected regions were generally above 0.6.

### 3.3. Spectral Data Pretreatments

**Figure 6:**
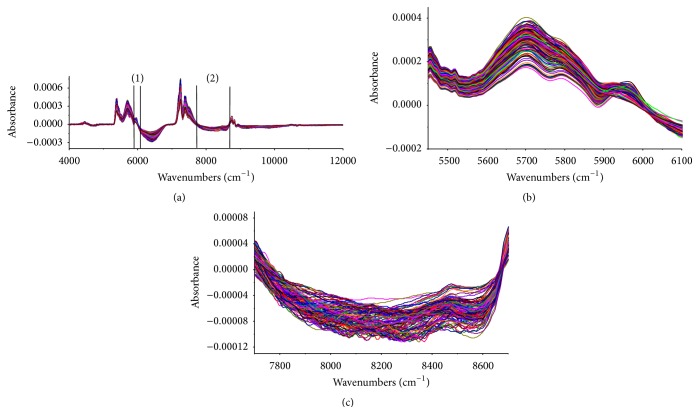
First derivative spectra with a 17-point Savitzky-Golay smoothing pretreatment. (a) The first derivative spectra with a 17-point Savitzky-Golay smoothing pretreatment from 4000 to 12000 cm^−1^; (b) the wavenumbers (1) from 5450 to 6100 cm^−1^; (c) the wavenumbers (2) from 7700 to 8700 cm^−1^.

Different spectral pretreatments were investigated to optimize the calibration performance. Several preprocessing methods were tested with the NIR spectra, including VN, MSC, constant offset elimination, first derivative, SLS, and their combinations. The VN and MSC are commonly used to eliminate irrelevant information in the spectra from unknown sources such as surface irregularities, distance variation of sample, and detector. The SNV transformation was applied to correct for light scattering and to reduce the changes in the path length. The 1st Der can reduce peak overlap and eliminate constant and linear baseline drifts. The SLS corrected the baseline. The detailed description of the techniques can be found in [40, 41]. During the water precipitation process, the flow rate influences the spectral baseline, and derivatives not only reduce peak overlap, but also eliminate linear baseline drifts [42]. However, the second derivative operation decreased the signal-to-noise ratio. Therefore, the first derivative was used to eliminate the spectral differences from baseline shifts. To avoid enhancing the noise, all of the derivative spectra were smoothed with a 17-point Savitzky-Golay filter [43]. The first derivative spectra with a 17-point Savitzky-Golay smoothing pretreatment, obviously reflected the quality attributes investigated in the 5450–6100 cm^−1^ and 7700–8700 cm^−1^ regions. The pretreated spectra were depicted in Figure 6.  Figure 6(a) showed the first derivative spectra with a 17-point Savitzky-Golay smoothing pretreatment from 4000 to 12000 cm^−1^. The spectral regions of wavenumbers (1) and wavenumbers (2) in Figure 6(a) were from 5450 to 6100 cm^−1^ and 7700 to 8700 cm^−1^, respectively, as shown in the Figures 6(b) and 6(c).

Comparing several different preprocessing techniques for modeling (results summarized in Table 3) indicates the first derivative preprocessing method yielded the best DSS, PA, RA, and SAB models.

### 3.4. Establishing the Calibration Models

**Figure 7:**
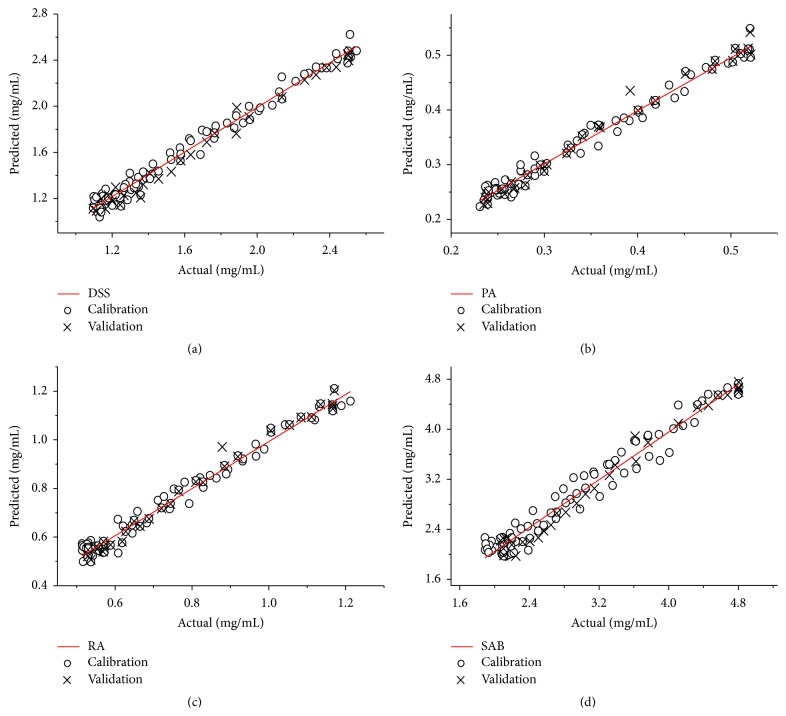
Calibration models for DSS (a), PA (b), RA (c), and SAB (d).

Four calibration models were established using the first derivative pretreatment in the regions from 5450 to 6100 cm^−1^ and 7700 to 8700 cm^−1^. The regression plots between the measured and predicted values using HPLC and NIR, respectively, for the DSS, PA, RA, and SAB concentrations in calibration and validation sets are depicted in Figure 7. Based on the data in Table 3, the *R* ^2^, RMSECV, and RMSEC values for the four first derivative pretreatment models were superior to the spectra using other preprocessing methods. Parameters of selected NIR models for DSS, PA, RA, and SAB were shown in Table 4. The *R* ^2^ values were all above 0.96. The calculated RSEC values were 3.71%, 3.72%, 3.91%, and 5.72% for DSS, PA, RA, and SAB, respectively. The RPD values were 7.49, 7.34, 7.18, and 5.06 for DSS, PA, RA, and SAB, respectively. Generally, RPD values above three are considered good for prediction purposes [44, 45]. Based on the above results, the four models were reliable and could accurately predict the quality attributes, and the water precipitation process can be monitored via on-line NIR using the developed quantitative models.

### 3.5. On-Line Quantitative Monitoring

The established NIR models were used to monitor the water precipitation process and predict the on-line DSS, PA, RA, and SAB concentrations in real-time. The RSEP and RPD values were used to assess the accuracy of the predicted results. If the RSEP value was below 20% and the RPD value was above 3.0, the established model accuracy was acceptable [46].

**Figure 8:**
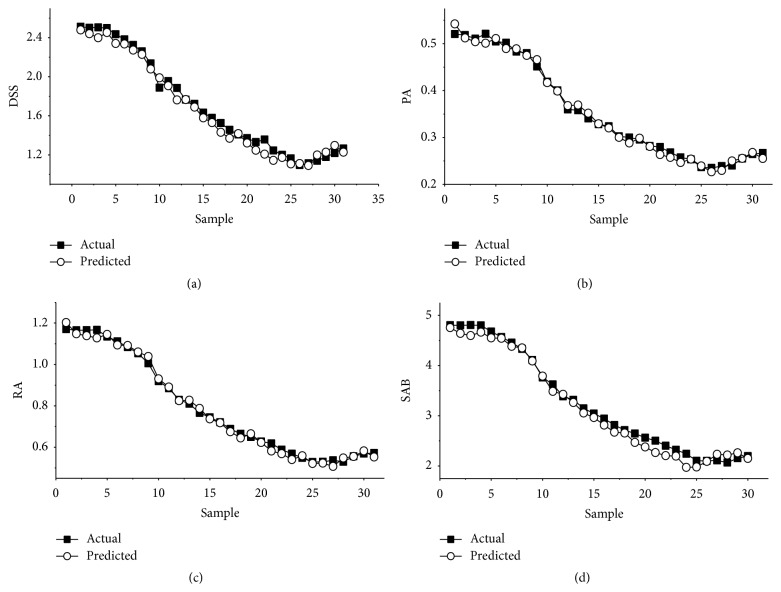
Predicted values for DSS (a), PA (b), RA (c), and SAB (d) obtained using the developed models.

To validate the applicability, the fifth sample batch (the prediction set) was scanned on-line and predicted using the established models.  Figure 8 shows the concentration trends predicted by the NIR in real-time and those obtained from the reference assay agreed well. In addition, the RSEP and RPD values (presented in Table 4) were calculated as 3.86% and 8.83 for DSS, 3.26% and 8.39 for PA, 3.02% and 9.25 for RA, and 4.02% and 8.22 for SAB, respectively. Validated results with RSEP values below 10% and RPD values above 3.0 met the practical requirements. All of these data demonstrated the developed models had high predictive accuracy during the on-line monitoring DSS, PA, RA, and SAB for the water precipitation process in Danhong injection. Hence, NIR spectroscopy is a suitable on-line quality control analysis technology for practical applications.

## 4. Conclusions

The present study has demonstrated that NIR spectroscopy provided us with important advantages to determine the concentrations of DSS, PA, RA, and SAB in a quantitative and nondestructive manner and in an extremely short time. Based on the HPLC reference method, the combination of NIR spectroscopy with PLSR was investigated for on-line detection of the compositions of Danhong injection during the water precipitation process. When the established models were used for prediction, these excellent results proved that NIR spectroscopy was valuable for on-line detecting the concentrations of DSS, PA, RA, and SAB. Finally, application of on-line NIR spectroscopy to Danhong injection production enabled us to detect, in real-time, changes in the compositions of Danhong injection during the water precipitation process.

Generally, industrial on-line applications have only rarely been described in scientific publications, although there is a need of further large-scale on-line studies in the pharmaceutical industry to verify reliability and accuracy of NIR spectroscopy under process conditions. However, many of the published studies appear to prove the suitability of NIR spectroscopy as a valuable tool for on-line process control and quality management or at least underline its potential for industrial applications.

## Figures and Tables

**Table 1:** HPLC method validation for DSS, PA, RA, and SAB.

Ingredients	Regression equations	*R*	Range (*μ*g/mL)	RSD of repeatability	RSD of stability	Average recoveries
DSS (mg/mL)	*y* = 3.241*x* − 1.152	0.9999	9.84~147.6	0.18%	0.88%	101.2%
PA (mg/mL)	*y* = 22.697*x* − 2.542	0.9999	1.13~22.68	1.08%	0.38%	98.2%
RA (mg/mL)	*y* = 8.554*x* − 1.459	0.9999	4.83~57.98	0.46%	0.60%	99.2%
SAB (mg/mL)	*y* = 5.984*x* − 64.335	0.9999	52.80~1056	0.10%	0.17%	100.8%

**Table 2:** DSS, PA, RA, and SAB concentrations for the final water precipitation (mg/mL).

Ingredients	Batches					Average	RSD (%)
	Fist	Second	Third	Fourth	Fifth		
DSS (mg/mL)	1.45	1.32	1.26	1.27	1.29	1.28	2.19
PA (mg/mL)	0.257	0.272	0.248	0.252	0.267	0.260	4.54
RA (mg/mL)	0.541	0.582	0.541	0.560	0.572	0.564	3.13
SAB (mg/mL)	2.71	2.22	1.93	1.95	2.19	2.07	7.35

**Table 3:** Parameters for the PLSR models with different spectral pretreatment methods.

Pretreatments	*R* ^2^				RMSEC (mg/mL)				RMSECV (mg/mL)			
	DSS	PA	RA	SAB	DSS	PA	RA	SAB	DSS	PA	RA	SAB
Raw spectra	98.08	97.81	97.76	95.97	0.0642	0.0141	0.0312	0.177	0.0684	0.0150	0.0333	0.1886
SLS	87.26	88.99	89.22	91.15	0.1540	0.0341	0.0716	0.263	0.1619	0.0356	0.0748	0.2765
VN	97.61	97.64	97.57	95.86	0.0716	0.0147	0.0340	0.180	0.0763	0.0157	0.0363	0.1905
1st Der	98.20	98.14	98.06	96.10	0.0619	0.0130	0.0304	0.174	0.0651	0.0137	0.0320	0.1806
2nd Der	81.38	80.63	83.88	85.45	0.2000	0.0420	0.0876	0.337	0.2024	0.0425	0.0887	0.3411
1st Der + SLS	89.20	89.58	90.79	88.84	0.1520	0.0308	0.0662	0.295	0.1577	0.0320	0.0687	0.3042

SLS: straight line subtraction.

VN: vector normalization.

1st Der: first derivative.

2nd Der: second derivative.

*R* ^2^: coefficient of determination.

RMSECV: root mean squares error of the cross-validation.

RMSEC: root mean square error of the calibration.

**Table 4:** Parameters of selected NIR models for DSS, PA, RA, and SAB.

Ingredients	*R* ^2^	RSEC	RPD of calibration	RSEP	RPD of prediction
DSS	0.9820	3.71%	7.49	3.86%	8.83
PA	0.9814	3.72%	7.34	3.26%	8.39
RA	0.9806	3.91%	7.18	3.02%	9.25
SAB	0.9610	5.72%	5.06	4.02%	8.22

## Abbreviations

NIR: Near-infrared
PLSR: Partial least squares regression
DSS: Danshensu
PA: Protocatechuic aldehyde
RA: Rosmarinic acid
SAB: Salvianolic acid B
RSEC: Relative standard errors of calibration
RSEP: Relative standard errors of prediction
RPD: Ratio of prediction to deviation
PAT: Process analytical technology
HPLC: High Performance Liquid Chromatography
*R*: Correlation coefficients
RSD: Relative standard deviation
SLS: Straight line subtraction
VN: Vector normalization
SNV: Standard normal variate
MSC: Multiplicative scatter correction
1st Der: First derivative
2nd Der: Second derivative
PLSR: Partial least squares regression
*R*^2^: Coefficient of determination
RMSEC: Root mean square errors of calibration
RMSEP: Root mean square errors of prediction
RMSECV: Root mean squares error of cross-validation.
